# Supplementary material for: Added Value of Viscoelasticity for MRI-Based Prediction of Ki-67 Expression of Hepatocellular Carcinoma Using a Deep Learning Combined Radiomics (DLCR) Model
Source: Cancers (Basel). 2022 May 24;14(11):2575. doi: 10.3390/cancers14112575 (PMC9179448; doi:10.3390/cancers14112575)
Supplement: Supplementary file 1 [file cancers-14-02575-s001.zip › cancers-1689214-supplementary.pdf]

Supplementary Material: Ki-67 Expression of Hepatocellular Carcinoma Using a Deep Learning Combined Radiomics (DLCR) Model

Xumei Hu, Jiahao Zhou, Yan Li, Yikun Wang, Jing Guo, Ingolf Sack, Weibo Chen, Fuhua Yan, Ruokun Li and Chengyan Wang

Table S1. Scan parameter of the multi-parametric MRI.

	Siemens					Philips				
**Variable**	**T2w**		**DWI**	**DCE**		**T2w**		**DWI**	**DCE**	
	Axial	Coronal	Axial	Axial	Coronal	Axial	Coronal	Axial	Axial	Coronal
TR (ms)	3000	1200	6600	4.85	3.90	2000	1100	1520	3.7	5.2
TE (ms)	95	91	62	2.41	1.49	66	80	70	1.32	1.07
Flip angle (degrees)	140	180	180	10	10	90	90	90	10	10
Field of view (mm)	380 × 380	380 × 380	380 × 306	380 × 3093	360 × 360	360 × 360	450 × 450	327 × 399	400 × 352	380 × 380
Matrix	320 × 320	256 × 256	134 × 134	20 × 240	320 × 234	288 × 288	320 × 282	108 × 128	268 × 234	212 × 212
Slice thickness (mm)	5	6	5	3	3	5	5	5	4	4.4

DWI = diffusion-weighted imaging; DCE = dynamic contrast-enhanced image.

Table S2. Parameter of the CNN networks.

Variable	Input Size	Layers	Conv Layers	Parameter Count
Inception-Resnet	299 × 299	-	-	60M
Inception	299 × 299	-	-	23.2M
Resnet	224 × 224	50	49	25.6M
Xception	299 × 299	-	36	22.8M
VGG16	224 × 224	16	13	>500M
VGG19	224 × 224	19	16	>500M

© 2022 by the authors. Licensee MDPI, Basel, Switzerland. This article is an open access article distributed under the terms and conditions of the Creative Commons Attribution (CC BY) license (http://creativecommons.org/licenses/by/4.0/).
